# Cellular Immunity-Pathogen Interactions in Infectious Diseases

**DOI:** 10.1155/2015/739783

**Published:** 2015-10-27

**Authors:** Muhammad Abubakar, Hasan T. Atmaca, Muhammad A. Zahoor, Oguz Kul

**Affiliations:** ^1^National Veterinary Laboratory, Islamabad 44000, Pakistan; ^2^Kirikkale University, 71000 Kirikkale, Turkey; ^3^McMaster Immunology Research Centre (MIRC), McMaster University, Hamilton, ON, Canada L8S 4L8

The interactions among host immunity, pathogens, and environment are ever-going process of disease and health. In nature, there is an ongoing equilibrium between infectious agents and their susceptible host organisms. While challenge of immune-competent individuals to low pathogenic strains usually results in elimination of the infectious agent, the other host-pathogen matches generally produce clinical courses of the disease. On the other hand, many pathogens produce certain enzymes, toxins, or proteins, which lead to the increased cellular adhesion and invasion abilities and also increased pathogenicity. However, in some instances, host innate immunity shows nonspecific resistance to such infectious diseases and it is called host resistance.

For example,* Bacillus anthracis* produces high mortality in its hosts, mammalian animals, including humans, but anthrax does not cause a fatal disease in the poultry. Otherwise, lineage 4 strain of the peste des petits ruminants which is prevalent in the Asian and Middle Eastern countries is responsible for the huge outbreaks among goat herds but not for sheep. These host resistance examples can be extended by host breed, age, and gender levels and the current knowledge has evidenced the presence of the disease resistance genes in the host genome.

The completion of full genome mapping in human and several animals allows the scientists to analyze host resistance genes against such a specific disease by the comparison of sick and healthy individuals' bioinformatics data. This means that the next discovery will be the definition of host resistance genes and their relations with cellular immunity in infectious diseases.

Keeping in view all these issues, we offered this special issue to the scientific community and we had an overwhelming response from them in form of 20 submissions including both research and review articles. Eight out of twenty submissions were accepted after a rigorous process of peer review.

This special issue incorporates four review papers and four original research papers. The review papers introduce concepts and themes underpinning the research papers including the immune invasion strategies by various pathogens including bacteria, virus, and parasite and also the interplay of host immune cells.

*Muhammad Abubakar* *Muhammad Abubakar* *Hasan T. Atmaca* *Hasan T. Atmaca* *Muhammad A. Zahoor* *Muhammad A. Zahoor* *Oguz Kul* *Oguz Kul*

